# Salmagundi

**Published:** 1886-06

**Authors:** 


### SALMAGUNDI.

EDITED BY E. G. BETTY, D.D.S.

PROFESSOR—Which teeth come last?
PUPIL—The false ones, sir.—*Clipping.*

DR. H. L. MOORE, of Cincinnati, has returned from Hot Springs, Ark., much improved in health.

JESSE FENRY, an armless man, exhibiting himself in the museums, handles a pen very skillfully with his teeth.

"IT is said that in China the fee for medical attendance is from five to ten cents a visit." What do dentists get?

A Chicago paper announces that Miss Adele Grant, a belle of New York, is engaged to be married to Lord Gumboil.

A sharp attack of catarrh, combined with the loss of the front teeth, seems to a foreign ear a prerequisite to the proper pronunciation of French.—*J. T. Perry.*

IT should not be forgotten that the Executive Committee of the Mississippi Valley Association is empowered to appoint delegates to the American Dental Association.

DR. J. R. CALLAHAN, of Hillsboro, has had the misfortune to lose his father, who died May 7th, aged over sixty years. The old gentleman was at one time a practicing physician.

DR. OTTO ARNOLD, formerly of Ironton, O., has recently removed to Columbus, where he has fitted up elegant quarters on High street. We wish him success in his new departure.

"Do you know how to count, my dear?" said an old gentleman to a little girl.

"Oh, yes, sir; I'll show you—one, two, three—you have three teeth."—*Graphic.*

---

It is stated that there is an old man residing on the Soquel road, Santa Cruz, Cal., who is at present cutting his third set of teeth. The process is attended with all the pain and annoyance with which a child suffers when it cuts its first teeth.

---

On Tuesday, May, 18th, the General Assembly of Ohio adjourned. The new Dental Law had been read before it, but, with hundreds of other bills, was laid over without action. It may be reached and acted upon when the Legislature re-assembles.

---

On Saturday afternoon, April 24th, Geo. W. Vanderbilt laid the corner stone of the new building for the College of Physicians and Surgeons, at Fifty-ninth street and Tenth Ave., New York. It will be known as the Vanderbilt Clinic, the family having given for the purpose a round million of hard dollars.

---

It appears to us that it is about time our dental Bible should be revised, and such terms as "six-year molar," etc., should be abolished and a proper nomenclature established that will be equally definitive and scientific, *i. e.*, according to Hoyle. One of the most absurd terms in use, constant at that, is, "artificial plate." Who ever heard of a natural one?

---

Bismarck is now discriminating against American dentists, who hereafter must practice in the Empire under a "trader's license" instead of under a "royal license," as heretofore. German dentists must be protected. Dentistry must be a lucrative business in a land where seven-cornered words are common. The remark of an American dentist to a German patient with a fractured jaw, "I think you must have been yawning over one of Goethe's comedies, or carelessly reading one of Klopstock's odes out loud," is supposed to have angered the Chancellor.—*Clipping.*

A MIXTURE of one part of iodoform to ten or fifteen of collodion, if spread repeatedly upon a neuralgic surface until it attains a thickness ot one to two millimetres, is said to be quite effective in the treatment of certain neuralgias. If the first application does not speedily terminate the neuralgia, those who have used this mode of treatment direct that its application should be continued. It seems especially valuable in the relief of neuralgias of the trigeminus. It also seems of value to be applied along the spine, particularly at painful points in what is called spinal irritation. These observations are by no means new, and yet they seem worthy of further consideration.—*Neurological Review.*

---

CINCINNATI, May 24th.

EDITOR SALMAGUNDI:

When the arch is at different points hard and soft, as is frequently met with, a difficulty is experienced in producing a satisfactory fit of the plate, owing to a faulty impression. I have overcome this by taking first a wax impression, and then after carefully examining the mouth to fix the hard points, cut away these corresponding points in the wax impression, and fill up with plaster, and introduce as in taking an original impression. The idea is to have the wax impression force the soft parts to a solid base, which will prevent trouble by a rocking motion which is seen to annoy and discourage those unfortunate enough to demand artificial teeth.

I have found this plan more satisfactory than the system of reliefs or building up the cast to correspond with the hard places in the mouth.

C. H. JAMES.

---

SALMAGUNDI spent a few days last month with her friend, Dr. D. R. Jennings, of Cleveland, at his beautiful home in the City by the Lake. The visit was not oniy a pleasant one, but full of profit as well, for Dr. Jennings is as full of "little tricks" as an egg is of meat. One of them is a matrix made of the thinnest possible triple plate tin. The tin is to be cut into the desirable size for any given case, and then bent into such shape as will exactly adapt itself to that face of the tooth upon which the cavity is located. The Doctor says there are fifty ways of making as many different matrices, as the tin is very malleable; a U shaped one for instance, inserted between two teeth, and a wedge driven in between the two surfaces of tin will force it into place against the two teeth, where there are contiguous cavities. Amongst other advantages, the reflection from the mirror-like surface of the matrix illuminates the cavity remarkably well, and that alone to one who mallets for himself, is an item of no little importance. Some other things we learned, we shall give our readers in a later issue.

